# Genome-Wide Analysis of the *MADS-box* Gene Family and Expression Analysis during Anther Development in *Salvia miltiorrhiza*

**DOI:** 10.3390/ijms241310937

**Published:** 2023-06-30

**Authors:** Songyue Chai, Kexin Li, Xuexue Deng, Long Wang, Yuanyuan Jiang, Jinqiu Liao, Ruiwu Yang, Li Zhang

**Affiliations:** 1 Featured Medicinal Plants Sharing and Service Platform of Sichuan Province, Sichuan Agricultural University, Ya’an 625014, China; chaisy@sicau.edu.cn (S.C.); 2020215019@stu.sicau.edu.cn (K.L.); x.x.deng@sicau.edu.cn (X.D.); longwangely@163.com (L.W.); 14273@sicau.edu.cn (Y.J.); liaojinqiu630@sicau.edu.cn (J.L.); yrwu@sicau.edu.cn (R.Y.); 2 College of Science, Sichuan Agricultural University, Ya’an 625014, China; 3 College of Life Sciences, Sichuan Agricultural University, Ya’an 625014, China

**Keywords:** *Salvia miltiorrhiza*, *MADS-box*, genome-wide, expression patterns, anther development

## Abstract

*MADS-box* genes constitute a large family of transcription factors that play important roles in plant growth and development. However, our understanding of *MADS-box* genes involved in anther development and male sterility in *Salvia miltiorrhiza* is still limited. In this study, 63 *MADS-box* genes were identified from the genome of the male sterility ecotype Sichuan *S. miltiorrhiza* (*S. miltiorrhiza*_SC) unevenly distributed among eight chromosomes. Phylogenetic analysis classified them into two types and 17 subfamilies. They contained 1 to 12 exons and 10 conserved motifs. Evolution analysis showed that segmental duplication was the main force for the expansion of the *SmMADS* gene family, and duplication gene pairs were under purifying selection. Cis-acting elements analysis demonstrated that the promoter of *SmMADS* genes contain numerous elements associated with plant growth and development, plant hormones, and stress response. RNA-seq showed that the expression levels of B-class and C-class *SmMADS* genes were highly expressed during anther development, with *SmMADS11* likely playing an important role in regulating anther development and male fertility in *S. miltiorrhiza*_SC. Overall, this study provides a comprehensive analysis of the *MADS-box* gene family in *S. miltiorrhiza*, shedding light on their potential role in anther development and male sterility.

## 1. Introduction

*MADS-box* (*Mini chromosome maintenance 1* (*MCM1*), *Agamous* (*AG*), *Deficiens* (*DEF*), and *Serum response factor* (*SRF*)) transcription factors constitute one of the largest families in plants [1]. They can be classified into two categories, type I and type II [2]. Type I *MADS-box* genes typically contain one to two exons and encode proteins with the highly conserved MADS-box (MADS) domain [3], but their biological functions remain largely unknown. In contrast, type II *MADS-box* genes contain six to eight exons [4] and have four typical domains: MADS, Intervening (I), Keratin-like (K), and C-terminal (C) domains [4,5]. The MADS domain enables DNA binding activity [6], while the I and K domains facilitate the formation of dimers and higher-order complexes between two or more MADS-box proteins [7,8,9], and the C domain contributes to transcriptional activation [6]. Due to this characteristic domain structure, type II genes are also known as MIKC-type *MADS-box* genes [5]. The *MADS-box* gene family has been identified and characterized in various plants, such as *Arabidopsis thaliana* (108) [10], *Oryza sativa* (75) [11], *Zea mays* (75) [12], *Vitis vinifera* (83) [13], and *Medicago sativa* (120) [14]. However, studies on *MADS-box* gene families in medicinal plants are limited.

*MADS-box* genes are crucial regulators of floral organ development [15], which encompass sepals, petals, stamens, and carpels [16]. The genetic control of floral organ development was elucidated by the ABC model, subsequently expanded by the ABCDE model [17]. Using *A. thaliana* as an example, sepal development is regulated by A-class (*APETALA1* (*AP1*)) and E-class (*SEPALLATA* (*SEP*)) genes, while petal development is regulated by A-class, B-class (*AP3* and *PISTILLATA* (*PI*)), and E-class genes. The formation of stamens is controlled by B-class, C-class (*AGAMOUS* (*AG*)), and E-class genes, while the development of carpels is governed by D-class (*SEEDSTICK* (*STK*) and *SHATTERPROOF* (*SHP*)) and E-class genes [17,18,19]. Many *MADS-box* genes have been reported to affect anther development and thus impact plant fertility. For example, loss of function of C-class *MADS-box* gene *OsMADS3* leads to brown anthers and a male-sterile phenotype in rice [20]. Overexpression of B-class *MADS-box* gene *BcAP3* causes abnormal development of the anther wall in *A. thaliana*, resulting in reduced pollen viability and ultimately leading to male sterility [21]. A recent study showed that overexpression of B-class *MADS-box* gene *PbTM6a* in tomato reduced pollen viability, and germination thus caused male sterility [22]. These highlight the importance of *MADS-box* genes in the regulation of male reproductive development in plants.

*Salvia miltiorrhiza* Bunge, commonly known as Danshen, is a perennial plant species native to China and has been used in traditional Chinese medicine for over 2000 years [23]. It is known for its medicinal properties and has been used to treat a variety of conditions, including cardiovascular disease, cancer, and various types of inflammation [24]. Moreover, due to its short life cycle, strong vitality, mature transgenic technology, small genome, and low chromosome numbers, *S. miltiorrhiza* is considered an ideal model medicinal plant [25,26,27]. Although *S. miltiorrhiza* is grown in many parts of China, there is a large variation in the yield of active ingredients among different areas, with the best production coming from Sichuan and Shandong province [28,29,30]. Notably, Sichuan ecotype (*S. miltiorrhiza*_SC) is rich in salvianolic acid B, while Shandong ecotype (*S. miltiorrhiza*_SD) is rich in tanshinone IIA [31,32]. Furthermore, the Sichuan ecotype exhibits male sterility [32,33]. However, it is still unclear whether *MADS-box* genes are involved in regulating the male sterility of *S. miltiorrhiza*.

In the present study, we identified *MADS-box* gene family members based on the *S. miltiorrhiza* (cv. Sichuan) genome data, and studied their phylogeny, gene structures, conserved motifs, gene duplication, collinearity, cis-acting elements, and interacting proteins. In addition, we investigated the gene expression profile of *SmMADS* genes at different anther developmental stages between two ecotypes of *S. miltiorrhiza* and identified differentially expressed genes (DEGs). The findings of our study will provide a comprehensive analysis of the *MADS-box* family members in *S. miltiorrhiza*_SC and shed light on their potential roles in regulating anther development and male sterility.

## 2. Results

### 2.1. Identification and Physicochemical Property Analysis of MADS-box Gene Famliy in S. miltiorrhiza_SC

Based on the hidden Markov model (HMM) of the SRF-TF domain (PF00319) and the K-box domain (PF01486), a total of 63 *MADS-box* family genes were found in S. *miltiorrhiza_SC* and named as *SmMADS1-63* based on their chromosomal and physical locations (Table 1). Protein physical and chemical properties, including the length of protein sequence, the molecular weight (MW), the isoelectric point (pI), and the subcellular localization, were analyzed (Table 1). Among the 63 *SmMADS* proteins, *SmMADS*48 was identified as the shortest protein with 66 amino acid (aa), whereas the longest one was *SmMADS*4 with 427 aa. The MW of the proteins ranged from 7.63 to 48.57 kDa, and the pI ranged between 4.90 (*SmMADS*4) and 10.15 (*SmMADS*23). Predictions of subcellular localization showed that all *SmMADS* proteins were located in the nucleus.

### 2.2. Phylogenetic Analysis of the MADS-box Gene Family

**Figure 1:**
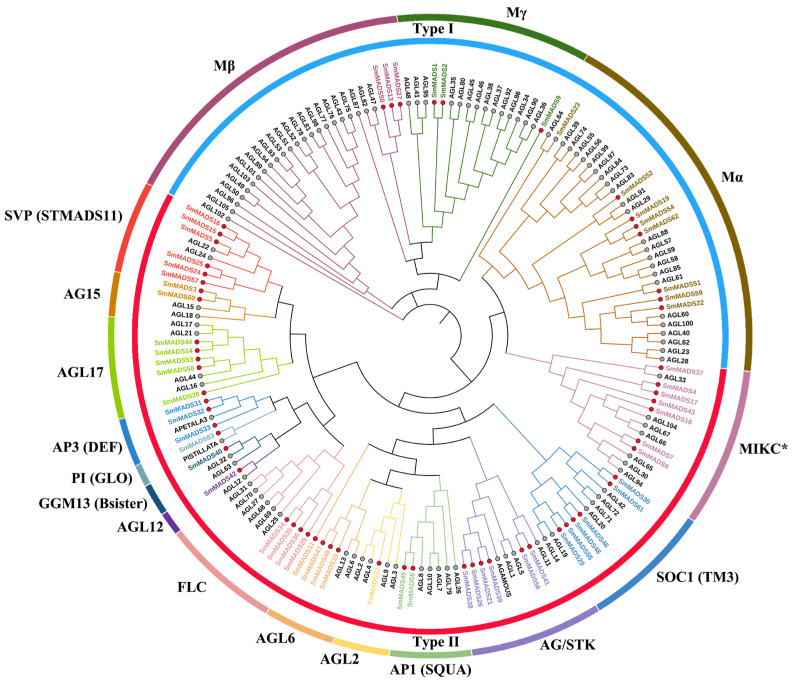
Phylogenetic tree of *S. miltiorrhiza_SC*-*A. thaliana MADS-box* transcription factors. Different subfamilies of proteins are highlighted with different colors. The red and gray circles represent *S. miltiorrhiza_SC* and *A. thaliana*, respectively.

A phylogenetic tree was constructed to clarify the evolutionary relationship of *MADS-box* proteins among 63 *SmMADS* and 108 AtMADS proteins. Based on the grouping of *MADS-box* family proteins in *A. thaliana* [10], the *MADS-box* proteins of *S. miltiorrhiza_SC* were classified into two types and 17 subfamilies (Figure 1). The type I (14) included three subfamilies: Mα (8), Mβ (3), and Mγ (3). The type II (49) included 14 subfamilies: MIKC* (7), AG/STK (6), AGL2 (1), AGL6 (4), AGL12 (1), AGL15 (2), AGL17 (5), AP1 (SQUA) (2), AP3 (DEF) (3), FLOWERING LOCUS C (FLC) (TM3) (4), GGM13 (Bsister) (1), PI (GLO) (1), SUPPRESSOR OF OVEREXPRESSION OF CONSTANS1 (SOC1) (6), and SHORT VEGETATIVE PHASE (SVP) (STMADS11) (6). Numbers in brackets represent the number of genes contained in each subfamily. Notably, AGL6, SVP, and AP3 subfamilies are significantly expanded in *S. miltiorrhiza_SC* compared with *A. thaliana*, and Mα, Mβ, Mγ, AGL2, and AP1 subfamilies are significantly contracted.

### 2.3. Chromosomal Distribution, Gene Structure, and Conserved Motif Analysis of SmMADS

**Figure 2:**
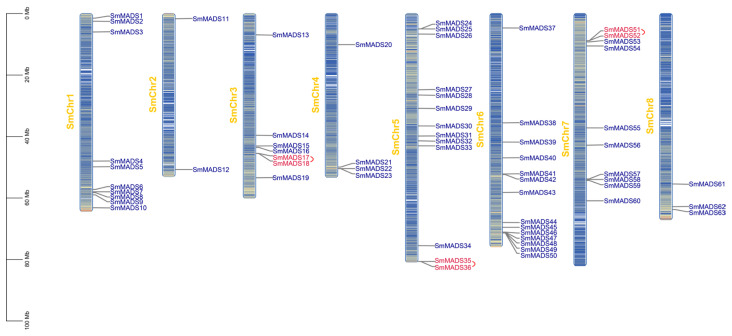
Chromosomal distribution of the *MADS-box* family members of *S. miltiorrhiza_SC*.

In total, *63 SmMADS* genes were unevenly spread over the eight chromosomes (Figure 2). Chromosome 6 had the maximum number of *SmMADS* genes (14), while chromosome 2 had the lowest number (2) (Table 1 and Figure 2). It can be seen that the majority of *SmMADS* genes were located at both ends of the chromosomes. 

**Figure 3:**
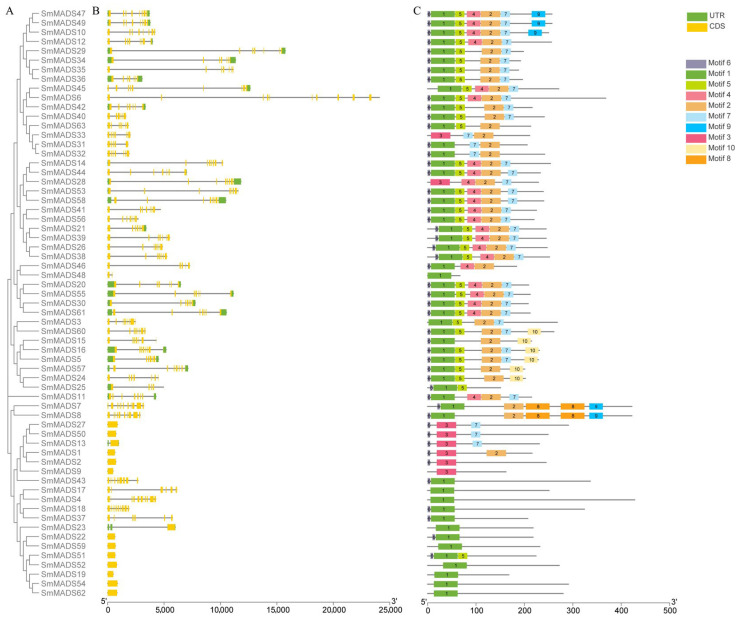
Gene structure and protein motif of *SmMADS* genes. (**A**) Phylogenetic tree of *SmMADS* proteins. (**B**) Gene structure of *SmMADS* genes. (**C**) Conserved motif of *SmMADS* proteins.

Gene structure analysis showed that the number of exons of *SmMADS* genes ranged from 1 to 12 (Table 1 and Figure 3A). Among type I *SmMADS* genes, 12 genes (*SmMADS*1/2/9/19/22/27/50/51/52/54/59/62) have only one exon and the other two genes *SmMADS*13 and *SmMADS*23 have two and three exons, respectively. Among type II *SmMADS* genes, except for *SmMADS*48 which has only two exons, the remaining 48 genes have 5 to 12 exons. It is obvious that the average number of exons in type II (7.6) is significantly higher than that in type I (1.2). Conserved motif analysis found a total of 10 conserved motifs, which are named motifs 1 to 10 (Figure 3B and Table S1). All *SmMADS* proteins had the conserved *MADS-box* domain (Motif 1 and 3) at the N-terminal, and K-domain (Motif 2, 4, and 7) was only found in type II *SmMADS* proteins.

### 2.4. Duplication and Synteny Analysis of SmMADS 

**Figure 4:**
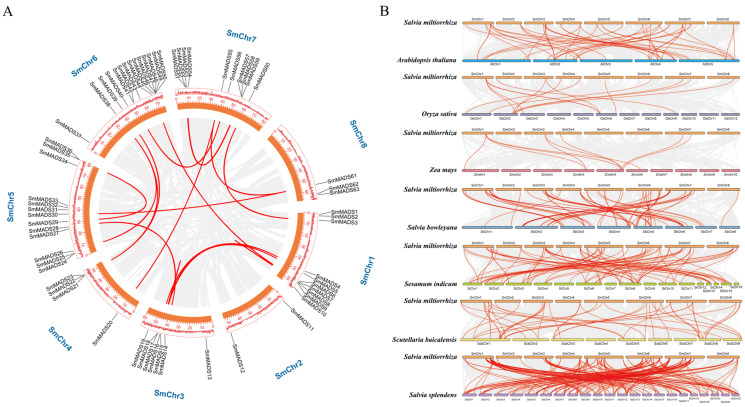
Synteny analysis of *MADS-box* genes between *S. miltiorrhiza_SC* and other plant species. (**A**) Synteny analysis of *MADS-box* genes in *S. miltiorrhiza_SC*. (**B**) Synteny analysis of *MADS-box* genes between *S. miltiorrhiza_SC* and *A. thaliana*, *O. sativa*, *Z. mays, S. bowleyana*, *S. indicum*, *S. baicalensis,* and *S. splendens*. The gray lines in the background indicate the collinear blocks within the genome, while the red lines highlight the syntenic of *MADS-box* gene pairs.

Gene duplication events mainly includes three categories: whole genome duplication (WGD), segmental duplication, and tandem duplication [34]. Among *SmMADS* genes, a total of six genes (9.52%) were found to form three tandem duplication gene pairs, and 23 genes (36.51%) formed 14 segmental duplication gene pairs (Figure 4 and Table S2). It can be seen that segmental duplication is the main force for the expansion of the *SmMADS* gene family. Then, we calculated Ka/Ks ratios to investigate the evolutionary pressures on the orthologous *MADS-box* gene pairs, we found that all gene pairs exhibited Ka/Ks <1 (Table S2), indicating that *SmMADS* genes were purified by selection and to mitigate harmful mutations. To further analyze the orthologous relationships between *SmMADS* genes and those of other species, seven species were subjected to synteny analysis, including monocotyledon plants: *O. sativa*, and *Z. mays*, dicotyledon plants: *A. thaliana*, *Sesamum indicum, Scutellaria baicalensis*, *S. bowleyana*, and *S. splendens*. A total of 445 pairs of orthologous genes were identified (Table S3). Among them, the highest number of collinear gene pairs (178) were found between *S. miltiorrhiza_SC* and *S. splendens*, followed by *S. baicalensis* (79), *S. bowleyana* (72), *A. thaliana* (48), *S. indicum* (45), *O. sativa* (14), and *Z. mays* (9). It can be assumed that the collinearity of *SmMADS* genes is more significant between dicotyledons. Furthermore, *SmMADS*4, *SmMADS*5, and *SmMADS*16 have collinear relationships with all seven species, which indicates that they have retained ancestral functions. 

### 2.5. Cis-Acting Elements Analysis of SmMADS

**Figure 5:**
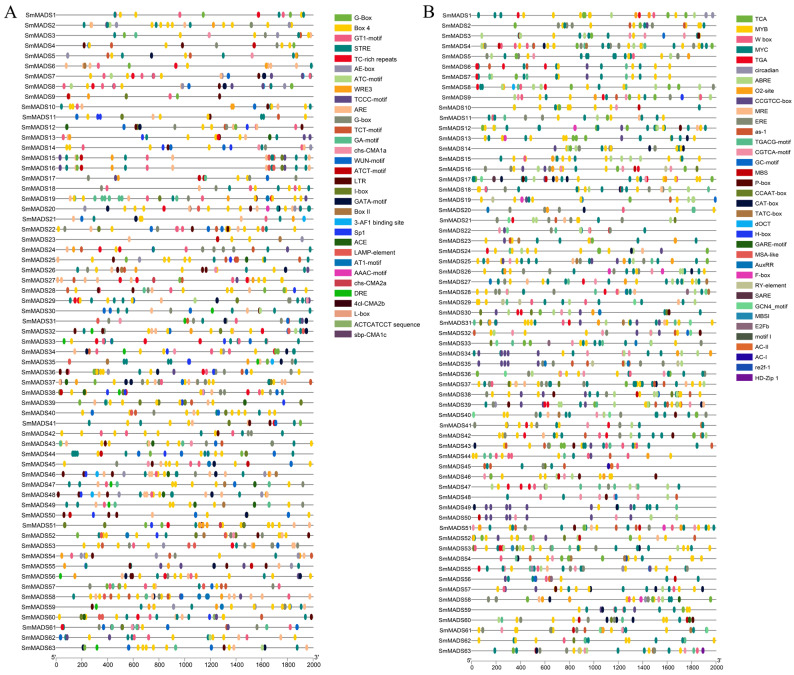
Predicted cis-elements in the promoter regions of the *SmMADS* genes. (**A**) Cis-elements related stress response. (**B**) Cis-elements related plant hormones and transcription factor binding sites.

To gain insights into the regulatory mechanisms of *SmMADS* gene expression, cis-acting elements were analyzed (Figure 5). The results revealed that many cis-acting elements related to plant growth and development, plant hormones, and stress response, we classified them into two types. Type I cis-acting elements are mainly related to stress response (Figure 5A), including light responsive elements (AT1-motif, ATC-motif, ATCT-motif, etc.), anaerobic responsive element (ARE), drought responsive element (DRE), low temperature responsive element (LTR), and wound responsive element (WRE3 and WUN-motif). Type II cis-acting elements are mainly related to plant hormones and transcription factor binding sites (Figure 5B), including salicylic acid responsive elements (as-1, TCA-element, and SARE), abscisic acid responsive elements (ABRE), auxin responsive elements (AuxRR and TGA), MeJA-responsive elements (CGTCA-motif and TGACG-motif), ethylene responsive elements (ERE), gibberellin responsive elements (F-box, GARE-motif, P-box, and TATC-box), MYB binding sites (MYB, CCAAT-box, MBS, and MBSI), MYC binding site (MYC), and HD-ZIP binding site (HD-Zip). 

### 2.6. Protein–Protein Interaction Network of SmMADS

**Figure 6:**
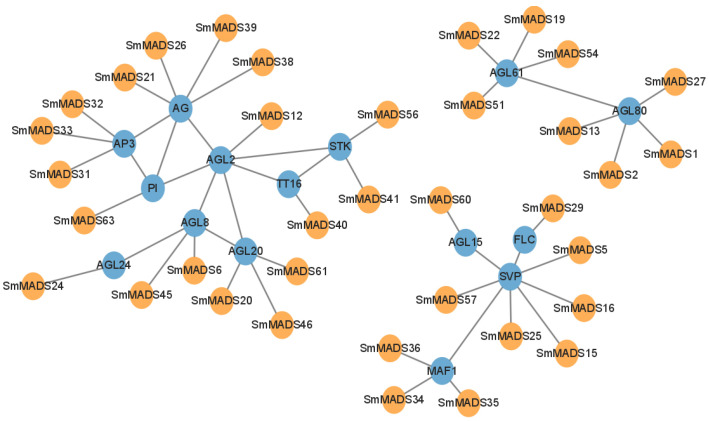
Protein–protein interaction network of *SmMADS* proteins based on their orthologs in *A. thaliana*.

To understand the interaction relationships and biological functions among *SmMADS* proteins, protein–protein interaction network was predicted based on *MADS-box* homologous gene in *A. thaliana*. A total of 36 *SmMADS* proteins homologous to those in *A. thaliana* and 15 corresponding interacting functional genes were identified (Figure 6). Among them, AG (*SmMADS*21/26/38/39), AP3 (*SmMADS*31/32/33), PI (*SmMADS*63), AGL2 (*SmMADS*12), AGL8 (*SmMADS*6/45), AGL20 (*SmMADS*20/46/61), AGL24 (*SmMADS*24), TT16 (*SmMADS*40), and STK (*SmMADS*41/56) might participate in flower development. SVP (*SmMADS*5/15/16/25/57), FLC (*SmMADS*29), MAF1 (*SmMADS*34/35/36), and AGL15 (*SmMADS*60) might participate in regulating flowering. AGL61 (*SmMADS*19/22/51/54) and AGL80 (*SmMADS*1/2/13/27) might participate in ovule development.

### 2.7. SmMADS Gene Expression during Anther Development

**Figure 7:**
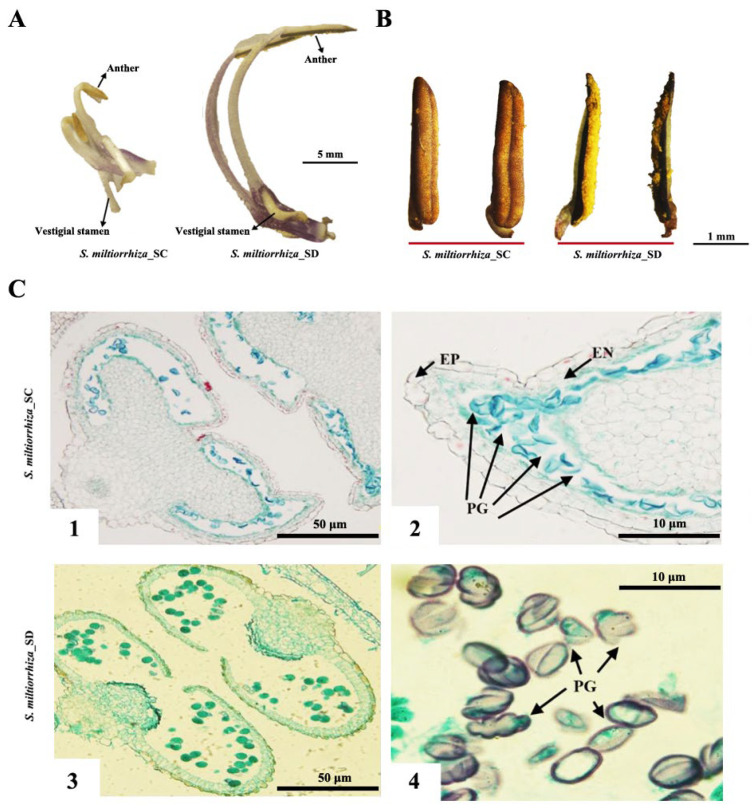
Morphologies of stamens and anthers of *S. miltiorrhiza_SC* and S. miltiorrhiza_SD at mature pollen stage. (**A**) Morphologies of stamens. (**B**) Morphologies of anthers. (**C**) Transverse sections of anthers; 1–2: *S. miltiorrhiza_SC*, 3–4: S. miltiorrhiza_SD, EP: epidermis, EN: drug chamber inner wall, PG: pollen grains.

*S. miltiorrhiza_SC* displays male sterility, with its stamens being shorter compared to those of S. miltiorrhiza_SD (Figure 7A). At the mature pollen stage, the anthers of S. miltiorrhiza_SD normally opened and released a large number of mature pollen grains, while those of *S. miltiorrhiza_SC* did not split or only released a very small amount of non-viable pollen grains (Figure 7B). Additionally, the pollen grains of S. miltiorrhiza_SD were fusiform or subspherical, whereas those of *S. miltiorrhiza_SC* tended to stick together and exhibited irregular shapes (Figure 7C). 

**Figure 8:**
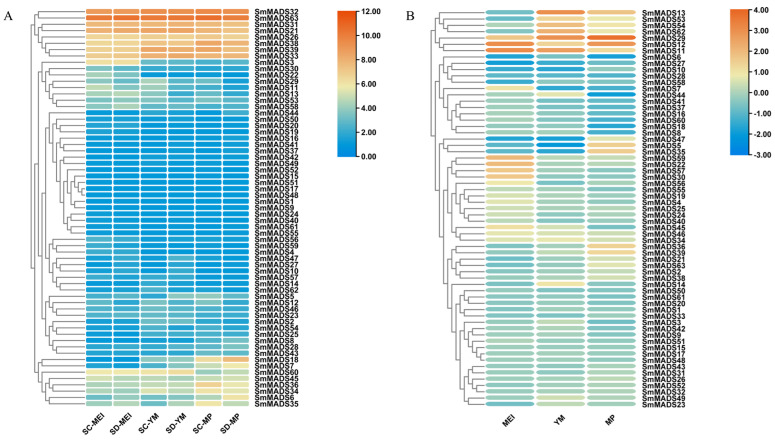
Expression profiles of *SmMADS* genes. (**A**) Expression profile of *SmMADS* genes in *S. miltiorrhiza_SC* and S. miltiorrhiza_SD at different stages of anther development. Gene expression level is shown on a graded color scale based on log_2_(FPKM + 1) values. (**B**) The ratio of the expression levels of *SmMADS* genes between *S. miltiorrhiza_SC* and S. miltiorrhiza_SD at different stages of anther development. The ratio of the expression levels is presented as log_2_ fold change (log_2_ FC) of the mean FPKM. SC: Sichuan; SD: Shandong; MEI: meiosis stage; YM: young microspore stage; MP: mature pollen stage.

In order to investigate the potential role of *SmMADS* genes in anther development and male sterility, we selected the anther of *S. miltiorrhiza_SC* and S. miltiorrhiza_SD (fertile) at three different developmental stages for RNA-seq. The transcriptome data of *SmMADS* genes showed high correlation between each replicate (Figure S1). Based on the FPKM data, we found that the expression level of type II *SmMADS* genes are significantly higher than type I (Table S4 and Figure 8A). It is worth noting that genes of AP3 (*SmMADS*31/32/33), PI (*SmMADS*63), and AG (*SmMADS*21/26/38/39) subfamilies were all highly expressed during anther development in two ecotypes of S. miltiorrhiza. Interestingly, these genes all belonged to the B-class (AP3/PI) and C-class (AG) *MADS-box* genes according to the ABCDE model [18].

We also analyzed DEGs in the two ecotypes of S. miltiorrhiza at three different anther developmental stages (Figure 8B). Significant differential expression (|log_2_ Fold change| > 1) of 25 *SmMADS* genes (3 genes at MEI stage, 11 genes at YM stage, and 11 genes at MP stage) was observed in *S. miltiorrhiza_SC* as compared to S. miltiorrhiza_SD. Among them, *SmMADS*11/12 were upregulated and *SmMADS*27 was downregulated at MEI stage, *SmMADS*11/13/29/54/62 were upregulated and *SmMADS*5/7/10/27/35/47 were downregulated at YM stage, and *SmMADS*5/11/12/13/29/35/36/39 were upregulated and *SmMADS*6/18/44 were downregulated at MP stage. 

## 3. Discussion

### 3.1. Identification and Classification of MADS-box Genes in S. miltiorrhiza_SC

With the completion of whole genome sequencing, the *MADS-box* gene family has been extensively studied in various plants, such as *A. thaliana* (108) [10], rice (75) [11], maize (75) [12], grape (83) [13], alfalfa (120) [14], cucumber (43) [35], lettuce (82) [36], and pear (95) [37]. In this study, 63 *SmMADS* genes were identified in the male sterility ecotype *S. miltiorrhiza*_SC, which is different from the 72 genes found in the male fertility ecotype *S. miltiorrhiza*_SD [38]. They were both divided into two types and 17 subfamilies according to phylogenetic relationship. Similar to many plants, such as rice, grape, lettuce, and pear [11,13,36,37], the number of type II genes was higher than type I. The gene structure analysis showed that the number of introns in type II genes was significantly higher than type I. As introns are involved in various steps of mRNA processing, including transcription, translation, and mRNA decay, they can have a significant impact on gene expression [39,40]. Thus, the regulating mechanism of type II genes may be more complex than type I. This phenomenon was also found in *A. thaliana*, rice, and pear [11,12,37]. Moreover, protein motif analysis showed that all *SmMADS* proteins had the highly conserved MADS domain, whereas type II genes contained unique I, K, and C domains, facilitating the formation of dimers and higher-order complexes between *MADS-box* proteins and transcriptional activation [6,7,8,9]. Therefore, type II *SmMADS* proteins have more complex protein structures, which also suggests that their regulatory mechanism may be more complex than type I. Additionally, we identified cis-acting elements in the promoter region of *SmMADS* genes that are associated with plant growth and development, plant hormones, and stress response, which was not analyzed in *S. miltiorrhiza*_SD [38]. These cis-acting elements play a crucial role in regulating the expression of related genes [40], enhancing the ability of *S. miltiorrhiza* to adapt to various adverse environments and ensure its normal growth and development. 

### 3.2. Evolutionary Analysis of SmMADS Gene Family

Gene duplication events play an important role in organismal evolution, which provides a genetic basis for the emergence of new traits [41,42,43]. These events mainly include WGD, segmental duplication, and tandem duplication [34]. In this study, 17 duplication gene pairs were found in *SmMADS* genes, and the majority of these pairs (82.35%) were found to be segmental duplications, which was lower than *S. miltiorrhiza*_SD (19 and 84.21%) [38]. This indicates that segmental duplication is the main force for the expansion of the *SmMADS* gene family, which is consistent with findings in other species, such as *Fagopyrum tataricum* [44], *Rhododendron hainanense* Merr. [45], and *Sechium edule* [46]. The WGD, as the driving force of the expansion of gene families [47], was not identified in the *SmMADS* gene family, which might be the reason for the relatively small number of *SmMADS* genes. Moreover, we calculated the Ka/Ks ratio between these duplicated gene pairs and found that all ratios were all less than 1, which suggested that these duplication gene pairs were under purifying selection, which reduces genetic diversity [41]. This observation also implies that they are relatively conserved and less diverged. Purifying selection typically contributed to the functional redundancy. Our results demonstrate that these duplication gene pairs have similar conserved motifs and gene expression patterns, which indicates that their function may therefore be redundant. These findings suggest that the *MADS-box* family is highly conserved in the evolution of *S. miltiorrhiza*. Synteny analysis is a powerful tool for understanding the evolutionary trajectory of genes [48]. In this study, some collinear gene pairs were only identified between *S. miltiorrhiza*_SC and dicotyledon plants, indicating that these homologous pairs were formed after the differentiation of dicotyledonous and monocotyledonous plants. Additionally, some collinear gene pairs were identified between *S. miltiorrhiza*_SC and all seven other species, indicating that these homologous pairs may have existed before the divergence of their common ancestor. These phenomena were also observed in *S. miltiorrhiza*_SD, although the analysis was limited to the collinearity between *S. miltiorrhiza_SD*, *A. thaliana*, and rice [38]. In summary, these results indicate that the *MADS-box* gene family in *S. miltiorrhiza* exhibits evolutionary conservation.

### 3.3. MADS-box Genes May Participate in Regulating the Anther Development and Male Fertility of S. miltiorrhiza 

The anther, together with the filament, constitutes the male reproductive organs of flowering plants and is where pollen development occurs [49]. The morphology and development of the anther are closely associated with the fertility of plants [50]. Previous studies have shown that *MADS-box* genes are crucial in anther development and male fertility. For example, the mutation of B-class and C-class *MADS-box* genes, such as *OsMADS3* [20], *BcAP3* [21], *PbTM6a* [22], *FaTM6* [51], and *OsMADS58* [52], can cause abnormal anther development and lead to male infertility. Gene expression profiling is important for determining gene function and biological processes. Most of the B-class and C-class *MADS-box* genes exhibited high expression levels in the stamen of *S. miltiorrhiza*_SD, indicating their significant roles in stamen development [38]. Our study demonstrated that all B-class (*SmMADS31/32/33/63*) and C-class (*SmMADS21/26/38/39*) genes were highly expressed during anther development in two ecotypes of *S. miltiorrhiza*, suggesting their crucial roles in anther development. Protein–protein interaction network revealed that there is an interaction between the B-class and C-class *SmMADS* proteins, suggesting that they do not act independently but form a complex to co-regulate the development of anthers. 

Comparing DEGs in the transcriptomes of normal and male-sterile anthers is of great significance for identifying key genes underlying male sterility. In this study, *SmMADS11*, which belongs to the *AGL6-like* subfamily, was differentially expressed at all three anther development stages. Although not included in the traditional ABCDE model, *AGL6-like* genes play an important role in floral organ development [53]. For example, *AGL6-like* gene *AGL13* is involved in regulating the formation of male and female gametophytes in *A. thaliana* [54]. In maize and rice, *AGL6-like* genes *ZAG3* and *OsMADS6* both regulate the development of stamens and ovules [55,56]. In wheat, RNAi of *AGL6* results in the abnormal development of stamens and ovules [57]. Therefore, differential expression of *SmMADS11* in anthers may affect normal anther development and result in male sterility. We will investigate the functional aspects of this gene in a further study.

Overall, the above findings provide insight into the potential functional roles of *SmMADS* genes in regulating the anther development and male fertility of *S. miltiorrhiza*. 

## 4. Materials and Methods

### 4.1. Identification of MADS-box Genes in S. miltiorrhiza_SC

The genomic data of Sichuan *S. miltiorrhiza* (*S. miltiorrhiza*_SC) were published by our team and were publicly accessible at NCBI (https://www.ncbi.nlm.nih.gov/, accessed on 27 July 2022). The HMM profiles of the SRF-TF domain and the K-box domain were downloaded from the Pfam database (http://pfam.xfam.org/search, accessed on 11 January 2022). The HMMER version 3.3.1 software (http://hmmer.org/download.html, accessed on 12 January 2022) was utilized to identify the *MADS-box* gene family (E-value ≤ 10–10) [58]. The SMART program (http://smart.embl-heidelberg.de/, accessed on 20 January 2022) and Plant TFDB (http://planttfdb.gao-lab.org/index.php, accessed on 22 January 2022) were used to further ensure the accuracy of the screening results. In addition, physicochemical properties of *SmMADS* proteins, including the physical location, the molecular weights (MW), and theoretical isoelectric points (pI) were evaluated using the online ProtParam tool (http://web.expasy.org/protparam/, accessed on 11 August 2022). Subcellular localization prediction was carried out by using the Plant-mPloc server (http://www.csbio.sjtu.edu.cn/bioinf/plant-multi, accessed on 11 August 2022). 

### 4.2. Phylogenetic Analysis of SmMADS Genes

The *MADS-box* protein sequence of *A. thaliana* was downloaded from The Arabidopsis Information Resource (TAIR) database (http://www.arabidopsis.org/, accessed on 6 May 2021). Full-length amino acid sequences of the *MADS-box* protein of *S. miltiorrhiza_SC* and *A. thaliana* were aligned by Clustal W with default parameters [59]. A phylogenetic tree estimation under the maximum likelihood (ML) principle was constructed by MEGA X version 10.2.6 software with the neighbor-joining algorithm and default settings [60]. The evolutionary distances were calculated using the Poisson model. The phylogeny was tested by bootstrapping with 1000 replications. Finally, the ML tree was visualized by iTOL version 6 (https://itol.embl.de/, accessed on 12 March 2022) [61].

### 4.3. Chromosomal Localization, Gene Structure, and Conserved Motif Analysis

The distribution of *SmMADS* genes and gene density were extracted and visualized from the genome structure annotation (Gff file) using TBtools version 0.396 [62]. The exon–intron structure of *SmMADS* genes was constructed by the Gene Structure Display Server (GSDS) version 2.0 (http://gsds.cbi.pku.edu.cn/index.php, accessed on 21 March 2022) based on the Gff file [63]. MEME Suite version 5.5.0 (http://memesuite.org/tools/meme, accessed on 20 March 2022) [64] was employed to analyze the conserved motifs of *SmMADS* protein sequences. The maximum number of motifs was 10, the motif width ranged from 6 to 200 amino acid residues, and the screening threshold was E < e-10. Gene structure and conserved motifs were visualized by TBtools.

### 4.4. Duplication and Synteny Analysis 

Gene duplication analysis was performed by using Clustal W to compare coding sequences (CDS) of *SmMADS* genes. Gene duplication events were defined and included the following: the aligned region with a similarity above 75%, the length difference of sequences no more than 25%, and only 1 duplication event is counted for tightly linked genes. Multiple collinear scanning toolkit (MCScanX) [65] was utilized to analyze the collinear blocks of *MADS-box* genes across different species and visualized by TBtools. Synonymous (Ka) and nonsynonymous (Ks) substitutions, as well as their ratios, were calculated by KaKs_Calculator [66]. 

### 4.5. Cis-Element and Protein–Protein Interaction Network Analysis

The putative promoter sequence regions 2000 bp upstream of each *SmMADS* gene was extracted as the promoter sequence, cis-acting elements were predicted using PlantCARE (http://bioinformatics.psb.ugent.be/webtools/plantcare/html/, accessed on 2 April 2022) and visualized by TBtools. The *SmMADS* protein interaction network was examined using the online website String version 11.5 (https://string-db.org/, accessed on 12 August 2021) and visualized by Cytoscape version 3.9.1 software [67].

### 4.6. Plant Materials, Expression Profile Analysis of SmMADS Genes 

Stamens and anthers of *S. miltiorrhiza*_SC and *S. miltiorrhiza*_SD were collected from Zhongjiang, Sichuan Province. Paraffin sections were created according to Wang et al. (2015) [68]. In brief, the specimens were dehydrated using a graded ethanol series and embedded in paraffin (Hualing, Shanghai, China). The 5 µm thick cross-sections were placed on gelatin-coated slides (Solarbio, Beijing, China) and stained with safranine and fast green double dyeing (Solarbio, Beijing, China). Then, paraffin sections of anthers were observed using Olympus BX51 microscope (Olympus, Tokyo, Japan).

The RNA-seq data of anther of *S. miltiorrhiza*_SC and *S. miltiorrhiza*_SD at different anther developmental stages published by our team [33] (publicly accessible at NCBI, https://www.ncbi.nlm.nih.gov/, accessed on 28 July 2022) were used to explore the expression patterns and differentially expressed genes of *SmMADS* genes during meiosis stage (MEI), young microspore stage (YM), and mature pollen stage (MP). Gene expression levels were estimated using fragments per kilobase of transcript per million fragments sequenced (FRKM). Differential gene expression analysis between *S. miltiorrhiza*_SC and *S. miltiorrhiza*_SD was performed using the DEGSeq R package. A corrected *p*-value of 0.05 and |log2 (Fold change)| of 1 were set as the criteria for significant differential gene expression. The expression matrix was visualized using TBtools. 

## Figures and Tables

**Table 1:** Physicochemical properties of *SmMADS* proteins.

Gene Name	Gene ID	Gene Start	Gene End	Protein Length	MW (kDa)	pI	Exon	Subcellular Localization
*SmMADS1*	*SmiChr010159.1*	1,485,362	1,486,009	215	24.15	9.44	1	Nucleus
*SmMADS2*	*SmiChr010237.1*	2,485,729	2,486,463	244	26.91	8.78	1	Nucleus
*SmMADS3*	*SmiChr010463.1*	5,985,425	5,987,880	267	29.08	6.02	8	Nucleus
*SmMADS4*	*SmiChr012432.1*	47,986,871	47,991,143	427	48.57	4.90	11	Nucleus
*SmMADS5*	*SmiChr012518.1*	49,853,737	49,858,251	228	25.79	5.74	7	Nucleus
*SmMADS6*	*SmiChr013041.1*	57,213,037	57,237,118	367	41.27	8.76	11	Nucleus
*SmMADS7*	*SmiChr013114.1*	57,973,959	57,977,155	421	48.54	5.40	12	Nucleus
*SmMADS8*	*SmiChr013140.1*	58,250,724	58,253,616	421	48.19	5.24	10	Nucleus
*SmMADS9*	*SmiChr013188.1*	58,984,444	58,984,929	161	18.93	9.07	1	Nucleus
*SmMADS10*	*SmiChr013568.1*	63,166,262	63,170,460	249	28.74	8.82	9	Nucleus
*SmMADS11*	*SmiChr020206.1*	1,687,838	1,692,129	214	24.82	9.59	8	Nucleus
*SmMADS12*	*SmiChr022579.1*	50,749,433	50,753,421	255	29.21	6.71	8	Nucleus
*SmMADS13*	*SmiChr030457.1*	6,931,586	6,932,576	230	26.15	9.08	2	Nucleus
*SmMADS14*	*SmiChr032079.1*	39,590,711	39,600,916	253	29.35	9.23	8	Nucleus
*SmMADS15*	*SmiChr032342.1*	43,073,819	43,078,127	214	24.08	6.86	7	Nucleus
*SmMADS16*	*SmiChr032371.1*	43,524,700	43,529,892	230	25.94	6.94	8	Nucleus
*SmMADS17*	*SmiChr032483.1*	45,482,820	45,488,961	250	28.43	8.90	6	Nucleus
*SmMADS18*	*SmiChr032484.1*	45,496,314	45,498,200	323	36.59	5.38	11	Nucleus
*SmMADS19*	*SmiChr033147.1*	53,409,971	53,410,474	167	19.26	8.36	1	Nucleus
*SmMADS20*	*SmiChr040450.1*	10,096,933	10,103,415	208	23.97	9.10	7	Nucleus
*SmMADS21*	*SmiChr042485.1*	49,933,469	49,936,886	244	27.87	9.07	7	Nucleus
*SmMADS22*	*SmiChr042537.1*	50,422,600	50,423,253	217	24.31	6.40	1	Nucleus
*SmMADS23*	*SmiChr042539.1*	50,444,332	50,450,331	217	24.11	10.15	3	Nucleus
*SmMADS24*	*SmiChr050234.1*	4,973,889	4,978,379	201	22.73	5.49	7	Nucleus
*SmMADS25*	*SmiChr050236.1*	4,985,289	4,990,240	150	16.83	9.48	5	Nucleus
*SmMADS26*	*SmiChr050368.1*	6,663,001	6,667,860	246	28.40	9.57	7	Nucleus
*SmMADS27*	*SmiChr051941.1*	24,790,931	24,791,803	290	33.84	9.31	1	Nucleus
*SmMADS28*	*SmiChr052086.1*	26,494,694	26,506,514	228	26.32	8.26	8	Nucleus
*SmMADS29*	*SmiChr052419.1*	30,819,767	30,835,514	197	22.36	5.81	7	Nucleus
*SmMADS30*	*SmiChr052857.1*	36,559,133	36,566,908	207	24.32	9.41	7	Nucleus
*SmMADS31*	*SmiChr053058.1*	39,835,853	39,837,660	205	23.93	9.20	5	Nucleus
*SmMADS32*	*SmiChr053148.1*	41,413,138	41,415,040	241	28.18	9.60	7	Nucleus
*SmMADS33*	*SmiChr053244.1*	43,027,138	43,029,149	210	24.56	5.11	6	Nucleus
*SmMADS34*	*SmiChr054722.1*	75,497,647	75,508,993	191	21.78	6.76	7	Nucleus
*SmMADS35*	*SmiChr055132.1*	80,642,424	80,653,561	187	21.13	8.21	7	Nucleus
*SmMADS36*	*SmiChr055133.1*	80,660,607	80,663,672	195	22.45	8.20	7	Nucleus
*SmMADS37*	*SmiChr060209.1*	4,683,105	4,688,854	206	23.40	8.97	6	Nucleus
*SmMADS38*	*SmiChr061372.1*	35,548,758	35,554,001	251	29.43	9.08	7	Nucleus
*SmMADS39*	*SmiChr061672.1*	41,801,645	41,807,139	244	28.17	9.02	7	Nucleus
*SmMADS40*	*SmiChr061960.1*	46,937,821	46,939,442	240	28.00	6.84	6	Nucleus
*SmMADS41*	*SmiChr062294.1*	52,175,555	52,180,240	224	25.94	9.47	8	Nucleus
*SmMADS42*	*SmiChr062306.1*	52,366,574	52,369,925	215	24.68	8.28	7	Nucleus
*SmMADS43*	*SmiChr062755.1*	58,203,258	58,205,954	335	38.56	6.02	9	Nucleus
*SmMADS44*	*SmiChr063526.1*	68,122,359	68,129,359	232	26.77	9.21	7	Nucleus
*SmMADS45*	*SmiChr063695.1*	69,688,073	69,700,712	270	30.90	6.64	9	Nucleus
*SmMADS47*	*SmiChr063823.1*	71,111,091	71,114,807	256	28.85	7.75	8	Nucleus
*SmMADS48*	*SmiChr063826.1*	71,136,933	71,137,349	66	7.63	10.01	2	Nucleus
*SmMADS49*	*SmiChr063828.1*	71,151,764	71,155,538	256	28.85	7.75	8	Nucleus
*SmMADS50*	*SmiChr063850.1*	71,360,773	71,361,519	248	28.94	9.44	1	Nucleus
*SmMADS51*	*SmiChr070750.1*	8,813,120	8,813,791	223	24.89	9.34	1	Nucleus
*SmMADS52*	*SmiChr070751.1*	8,816,961	8,817,776	271	28.86	7.80	1	Nucleus
*SmMADS53*	*SmiChr070801.1*	9,225,527	9,237,091	238	27.38	8.87	8	Nucleus
*SmMADS54*	*SmiChr070926.1*	10,535,231	10,536,103	290	31.82	8.20	1	Nucleus
*SmMADS55*	*SmiChr073037.1*	37,186,399	37,197,551	211	24.10	9.22	7	Nucleus
*SmMADS56*	*SmiChr073378.1*	42,810,566	42,813,278	219	25.21	9.57	8	Nucleus
*SmMADS57*	*SmiChr074083.1*	53,784,259	53,791,389	200	22.81	9.66	9	Nucleus
*SmMADS58*	*SmiChr074093.1*	53,967,203	53,977,684	239	27.58	9.38	9	Nucleus
*SmMADS59*	*SmiChr074108.1*	54,295,919	54,296,614	231	25.54	5.75	1	Nucleus
*SmMADS60*	*SmiChr074429.1*	60,910,555	60,913,901	260	29.32	8.65	8	Nucleus
*SmMADS61*	*SmiChr082309.1*	55,443,038	55,453,573	211	24.18	9.49	8	Nucleus
*SmMADS62*	*SmiChr082932.1*	62,821,912	62,822,751	279	30.56	9.34	1	Nucleus
*SmMADS63*	*SmiChr083047.1*	63,750,692	63,752,525	212	24.93	6.51	7	Nucleus

MW: molecular weight, pI: isoelectric point.

## Data Availability

*S. miltiorrhiza* (cv. Sichuan) genome is available from the NCBI under project ID PRJNA862689. RNA-seq data are available from the NCBI under project ID PRJNA863332.

## Supplementary Materials

The following supporting information can be downloaded at https://www.mdpi.com/article/10.3390/ijms241310937/s1.
