# Supplementary figures and images for: Genome-Wide Analysis of the MADS-box Gene Family and Expression Analysis during Anther Development in Salvia miltiorrhiza
Source: Int J Mol Sci. 2023 Jun 30;24(13):10937. doi: 10.3390/ijms241310937 (PMC10341755; doi:10.3390/ijms241310937)

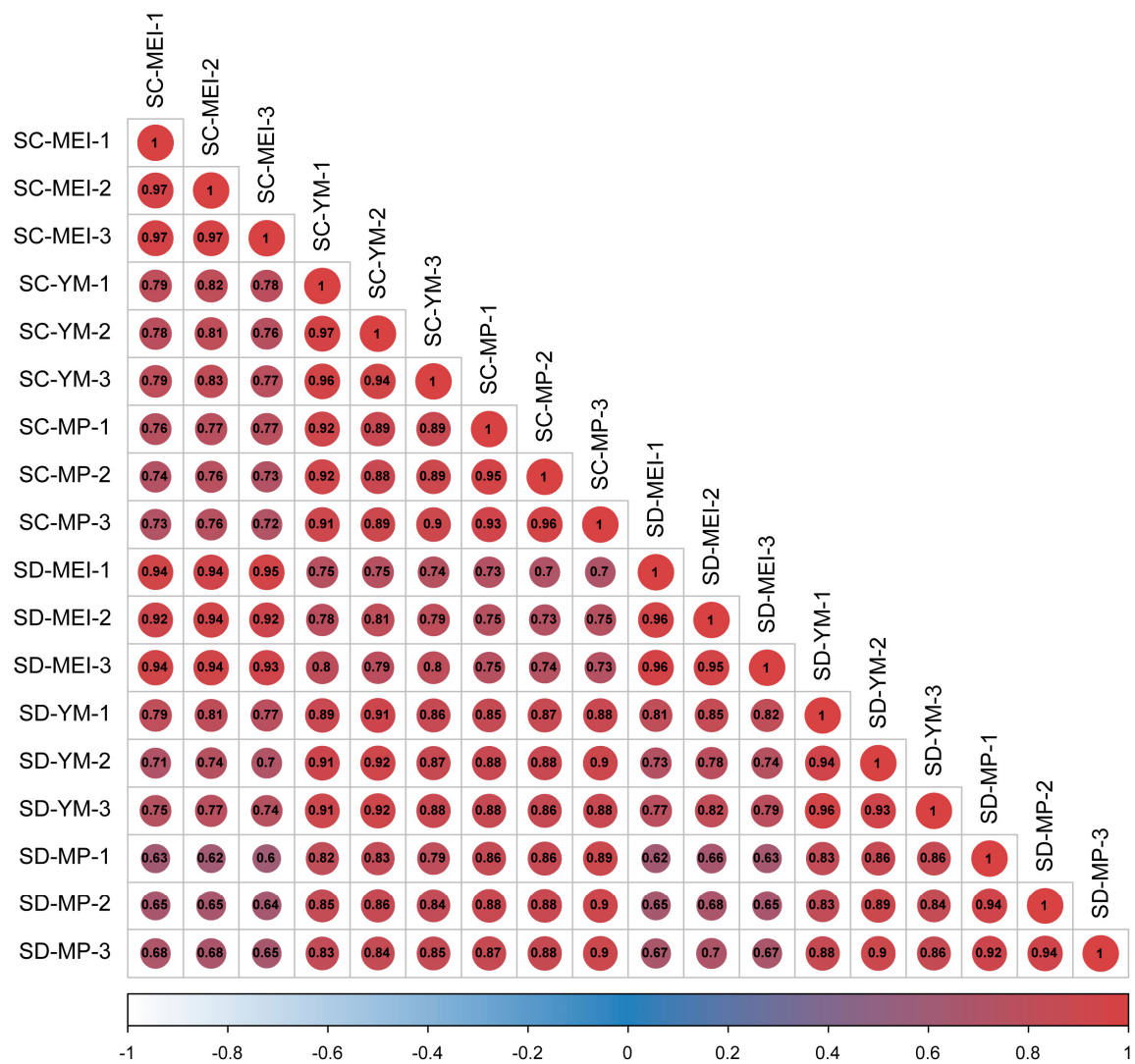

**Figure S1:** Correlation analysis between transcriptome data of each sample.

Supplement: Supplementary file 1 [file ijms-24-10937-s001.zip › Figure S1.pdf]
